# Endobronchial tuberculosis polyps

**DOI:** 10.1002/rcr2.595

**Published:** 2020-06-08

**Authors:** Christiaan Yu, Chuan Tai Foo, Ar Kar Aung, Simon A. Joosten

**Affiliations:** ^1^ Monash Lung and Sleep Monash Health Melbourne Victoria Australia; ^2^ School of Public Health and Preventative Medicine Monash University Melbourne Victoria Australia

**Keywords:** Endobronchial polyps, endobronchial tuberculosis, flexible bronchoscopy, tuberculosis

## Abstract

Endobronchial tuberculosis (TB) is an uncommon manifestation of *Mycobacterium tuberculosis.* We report a case of endobronchial TB polyps in a patient from India presenting with cough, loss of weight and night sweats. Computed tomography chest revealed enlarged mediastinal lymph nodes, endobronchial invasion, and nodular infiltrates in the right lower lobe. Flexible bronchoscopy revealed two endobronchial polyps at the carina and left main bronchus which were biopsied. Histopathology showed non‐caseating granulomas. Both the biopsy and bronchial washings did not identify acid‐fast bacilli on Ziehl‐Neelsen stain and had negative TB complex DNA polymerase chain reaction. One month after bronchoscopy, *M. tuberculosis* was cultured from the bronchial washings. Following six months of TB treatment, there was full resolution of symptoms and significant radiological improvement. We highlight the diagnostic challenges in endobronchial TB which may impact on the timely institution of treatment.

## Graphical abstract

We present a patient with endobronchial tuberculosis (TB) polyps for which initial acid‐fast bacilli staining and TB complex DNA polymerase chain reaction yielded negative results. Histopathology showed non‐caseating granulomatous inflammation and the diagnosis was ultimately achieved four weeks later when bronchial washing cultures yielded *Mycobacterium tuberculosis*, thus highlighting the challenges in achieving a timely diagnosis.

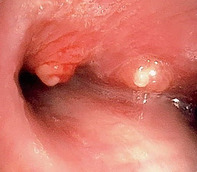

## Introduction

Endobronchial tuberculosis (TB) is an uncommon form of pulmonary TB. Its appearance in large airways may mimic cancer or sarcoidosis and biopsy findings can be non‐specific [1, 2]. This rare disease manifestation poses a diagnostic challenge, and further delays in management may lead to irreversible complications.

We present a patient with endobronchial TB polyps for which initial acid‐fast bacilli (AFB) staining and TB complex DNA polymerase chain reaction (PCR) yielded negative results. Histopathology showed non‐caseating granulomatous inflammation and the diagnosis was ultimately achieved four weeks later when bronchial washings cultures yielded *Mycobacterium tuberculosis*, thus highlighting the challenges in achieving a timely diagnosis.

## Case Report

A 22‐year‐old male was referred to hospital for evaluation of a two‐week history of intermittent fevers associated with a non‐productive cough, night sweats and weight loss. The patient was born in India and had arrived in Australia 12 months ago to study at a local university. He had no significant past medical history and never smoked. He did not recall any close contacts with TB.

On initial examination, his heart rate was regular at 80 beats/min, blood pressure 130/70 mmHg, respiratory rate 16 breaths/min and oxygen saturation of 97% on room air. He had a low‐grade temperature of 37.8°C. On clinical examination, there was no palpable lymphadenopathy, hepatosplenomegaly or skin lesions. His chest was clear and there was no wheeze. Initial blood tests showed a normal full blood count, renal and liver function tests. C‐reactive protein was mildly elevated at 36 mg/L. Human immunodeficiency virus serology was negative. Interferon gamma release assays for TB antigens were positive. He was unable to expectorate sputum for analysis.

**Figure 1:**
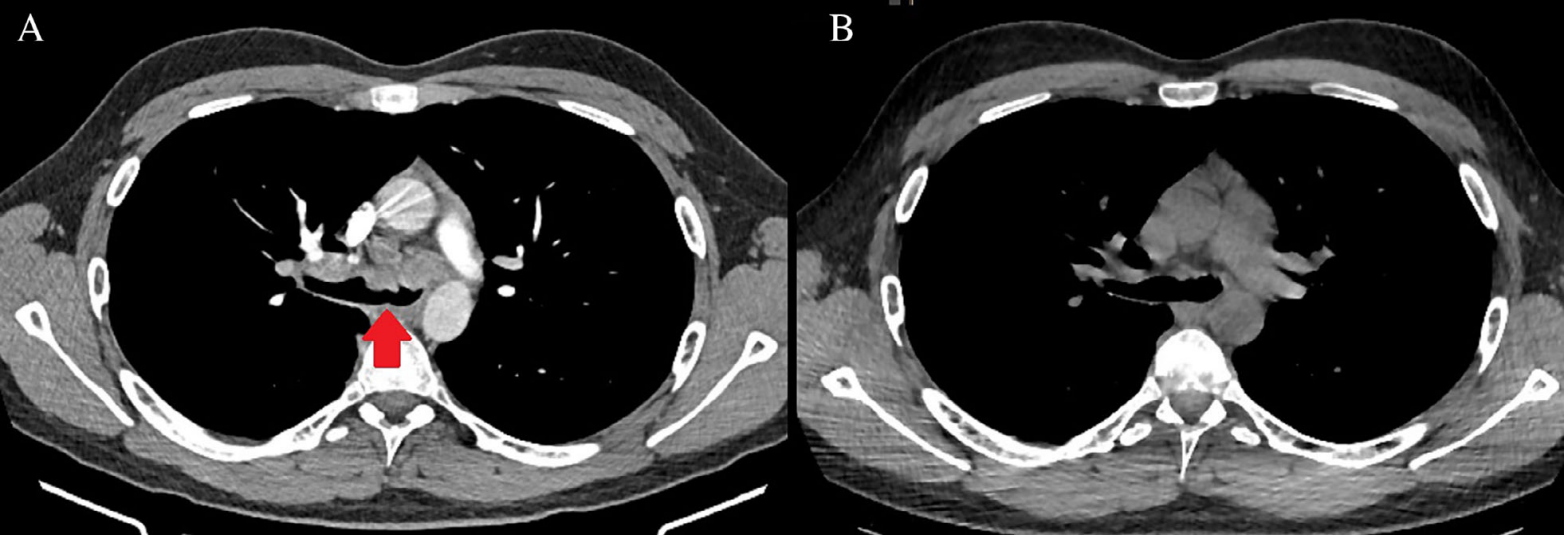
(A) Computed tomography (CT) chest demonstrating mediastinal lymphadenopathy and endobronchial involvement prior to tuberculosis (TB) treatment. (B) CT chest demonstrating significant improvement following six months of TB treatment.

Chest radiograph demonstrated a right paratracheal opacity with normal lung fields. Chest computed tomography (CT) revealed multiple enlarged mediastinal lymph nodes with endobronchial involvement (Fig. 1A) and multiple micronodular changes in the right lower lobe.

**Figure 2:**
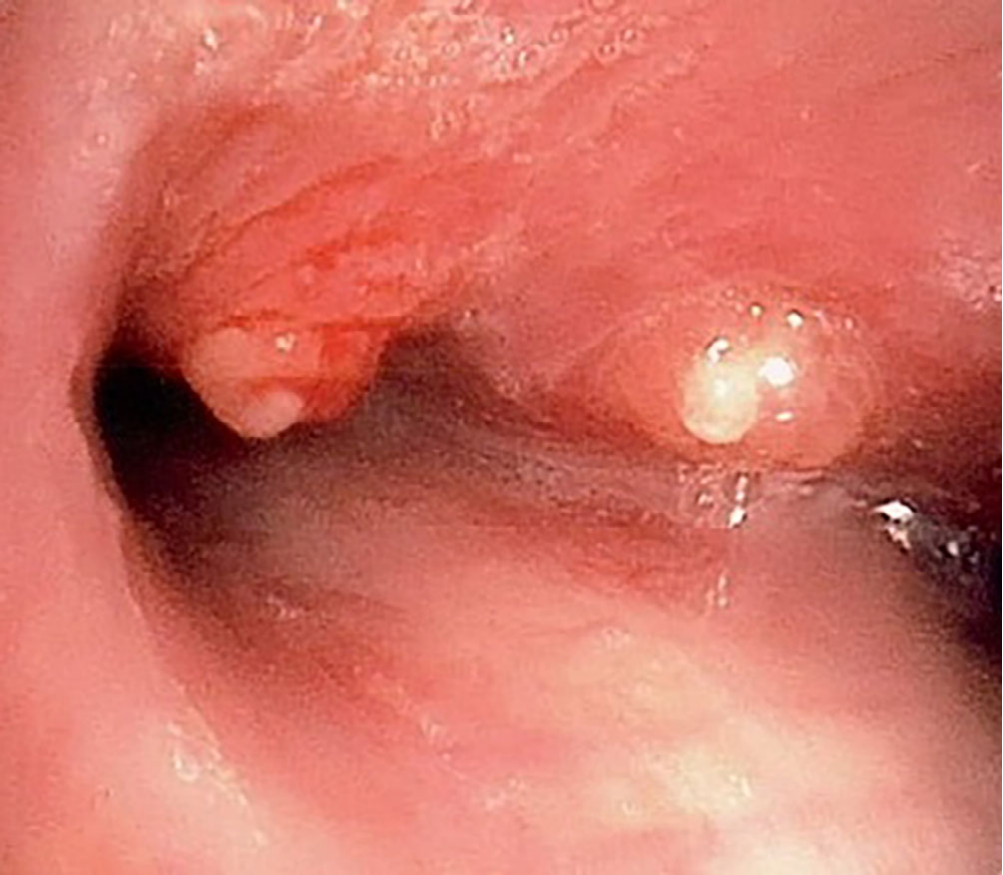
Endobronchial tuberculosis at the carina and left main bronchus visualized on flexible bronchoscopy.

Bronchoscopy was undertaken and showed two discrete endobronchial lesions; one at the level of the carina and the other at the orifice of the left main bronchus. The lesions were polypoid in nature and had normal surrounding mucosa (Fig. 2). They had mildly increased vascularity and were friable. Histopathology from forceps biopsies of these lesions showed non‐caseating granulomatous inflammation with predominant lymphocytic and plasma cell infiltrate. AFB smear and TB complex DNA PCR (GeneXpert MTB/RIF) of the biopsy sample were negative. Bronchial washings of the right lower lobe returned negative on AFB smear, TB complex DNA PCR and standard bacterial culture. There were no malignant cells and a low number of macrophages seen on microscopy which was insufficient for differential cell count.

The differential diagnoses included TB (with associated lymphadenitis and endobronchial component), sarcoidosis and malignancy. While awaiting results from TB culture, the patient was commenced on empirical treatment for pulmonary TB with rifampicin, isoniazid, ethambutol, pyrazinamide and pyridoxine as this was felt to be the most likely diagnosis. Four weeks later, *M. tuberculosis* was cultured from the bronchial specimens.

The patient completed six months of first‐line treatment for drug sensitive pulmonary TB with complete resolution of symptoms. Repeat chest CT showed significant improvement in thoracic lymphadenopathy with near complete resolution of the endobronchial lesions (Fig. 1B).

## Discussion

TB remains a health crisis in developing countries killing millions of people every year [3]. Global strategies and policies have been adopted to reduce the mortality and morbidity of this treatable disease [4]. Barriers to this include weak healthcare systems, resistance to anti‐TB drugs, and delays in diagnosis and treatment [5, 6, 7].

First described in 1939 following rigid bronchoscopy, endobronchial TB (EBTB) is a form of pulmonary TB involving the trachea and major bronchi [8]. This is estimated to affect up to 5.8% of patients with pulmonary TB [9], although rates as high as 50% have been reported by others [10]. Furthermore, EBTB is more likely to be encountered in TB endemic countries such as India and China, in comparison to Australia [11]. EBTB appears to be more common in younger females even though they have a lower incidence of pulmonary TB. Reasons for this include longer exposure to TB bacilli, as females are less likely to expectorate due to sociocultural reasons, and narrower bronchi anatomy compared to males [12]. This manifestation of TB poses a diagnostic and management challenge, especially in non‐TB endemic countries, as its clinical presentation, findings, and sequelae are highly variable [13].

Bronchoscopic appearance of EBTB is closely related to the underlying pathological process. To date, seven subtypes have been described, with oedematous‐hyperaemic, actively caseating, and granular subtypes being the most commonly reported [9]. The tumourous subtype, as seen in our case, is named after its tumour‐like appearance on bronchoscopy, and is thought to arise as a result of an inflammatory response involving the mucosal and submucosal layers of the bronchial wall by lymphocytes against mycobacteria [14]. Unlike the other subtypes, tumourous lesions are less common and have a highly unpredictable natural history necessitating closer monitoring [9].

Visually, the appearance of EBTB lesions can mimic sarcoidosis, malignancy, or granulomatous disease, misleading clinicians down alternate diagnostic pathways resulting in a delayed or incorrect diagnosis [15]. This has significant implications to the patient as untreated EBTB can lead to life‐threatening sepsis, tracheobronchial stenosis, distal airway collapse, fibrosis, and bronchiectasis [16].

Sputum AFB smears have a poor sensitivity in diagnosing pulmonary TB (as low as 45–50%) [17]. Furthermore, in EBTB sputum expectoration may be difficult due to mucus entrapment by proximal granulation tissue or endobronchial lesions [18]. Molecular tests such as TB DNA complex PCR test may have a greater sensitivity than smear microscopy, although this is highly dependent on where the sample was taken from [19]. Certain molecular tests such as GeneXpert MTB/RIF also have the ability to detect rifampicin‐resistance mutations. Ultimately conventional culture remains the most sensitive tool for detecting TB [20] but may take up to eight weeks due to the slow growth rate of mycobacteria. Endobronchial biopsies may assist in the diagnosis of EBTB if characteristic findings of caseating necrosis or AFB positive mycobacterium are identified. Nonetheless, biopsies from these lesions can also show non‐specific inflammation which may not assist in the diagnostic process [1, 2, 18]. Overall, diagnostic yields from both microbiological and histological examinations in EBTB have been reported to be poor in large case series [19]. Kim et al. have previously described the immense difficulties in diagnosing EBTB. In their report, the diagnosis was achieved close to two months after presentation following the third bronchoscopic sampling, with prior bronchial washings and endobronchial biopsies all returning negative for TB [21].

Even though our patient did not have any definite microbiological evidence of TB on preliminary results, given his clinical, radiological, and epidemiological risk factors, a decision was made to commence empiric TB treatment. TB culture returned positive four weeks later confirming the diagnosis. This obviated the need for further diagnostic procedures such as repeat flexible bronchoscopy with further sampling, rigid bronchoscopy with polypectomy, or in centres with endobronchial ultrasound capabilities, mediastinal lymph node sampling. All these alternatives carry their own risk and lengthen time to diagnosis.

We highlight the challenges in diagnosis of this condition and raise awareness of the need to consider TB as a differential for endobronchial lesions even in the absence of a positive AFB smear, TB DNA complex PCR and classic histological findings of caseating granulomas. Timely anti‐tuberculous therapy is vital in the eradication of, and minimization of transmission of TB, in addition to mitigating the risk of longer term complications of EBTB.

### Disclosure Statement

Appropriate written informed consent was obtained for publication of this case report and accompanying images.
